# Mood Swing during Menstruation: Confounding Factors and Drug Use

**DOI:** 10.4314/ejhs.v32i4.3

**Published:** 2022-07

**Authors:** Matthew Obaineh Ojezele, Anthony Taghogho Eduviere, Emmanuel Adesola Adedapo, Treasure Kurakughan Wool

**Affiliations:** 1 Department of Pharmacology, Delta State University, Abraka, Nigeria; 2 Department of Microbiology, Faculty of Science, University of Ibadan, Ibadan, Nigeria

**Keywords:** Mood Swing, Premenstrual Syndrome, Premenstrual Dysphoric Disorder, Bipolar Disorder, Menstruation, Endometriosis

## Abstract

**Background:**

Premenstrual syndrome (PMS) is a physiologic process in women where mood swing is one of the symptoms influencing the psycho-emotional, physical, and behavioral reactions exhibited by women during menstruation. This study elucidates the effect of mood swing, confounding factors and healthcare-seeking behaviors of women in an educational environment.

**Methods:**

Exactly 328 women who were within reproductive ages 16 and 35 years participated in this study. A survey method was adopted; validated and standardized questionnaires were administered to confidentially assess the effect of mood swing via PMS. All data were analyzed with SPSS 25.0; descriptive method was adopted and results were expressed in percentages.

**Results:**

Mood swing was discovered as a symptom overlapping with psycho-emotional, physical, and behavioral symptoms during menstruation. The overall PMS prevalence was 67.4% while PMDD prevalence was 25.6%. Psycho-emotional symptoms: anger, irritability, depression. Physical symptoms: coldness, paleness, food craving, breast tenderness, digestive changes. Behavioral symptoms: social withdrawal, nocturnal social activity, absenteeism, poor work or academic performance, increased libido. Confounding factors include stress, gynecological conditions such as endometriosis, uterine fibroid, ovarian cyst, pelvic adhesion, and polycystic ovarian syndrome. Also, 22.9% had a family history of bipolar disorder (BD) while 30.2% had previous diagnosis. Severe pain was a major factor for seeking treatment; Paracetamol, and Piroxicam were frequently used drugs.

**Conclusions:**

Severe PMS triggers mood swing and can badly affect academic or work activities; victims either endure the pain due to socio-cultural and financial factors or take unsuitable medications where abuse is inevitable.

## Introduction

Menstruation is a normal, repetitive, reproductive physiological process in women characterized by the shedding of the upper endometrium which begins at puberty and stops at menopause (1, 2). This process occurs in four main stages which are: follicular, ovulatory, luteal or premenstrual, and menstrual stages all of which is regulated by both nervous and endocrine systems (1). The luteal or premenstrual stage is usually accompanied with various symptoms which may be distressful and negatively affect the psycho-emotional, physical and behavioural output; this is referred to as premenstrual syndrome (PMS) while a severe PMS is referred to as premenstrual dysphoric disorder (PMDD). About 75% women complain of PMS, younger women less than 25 years, experience this syndrome than older women while PMDD occurs in 3–8% of women with PMS (2, 3). PMS accounts for 47.8% worldwide with higher prevalence in sub-Saharan Africa where Ethiopia reported 67% and 72% PMS prevalence respectively while 42.9% prevalence was reported in South-Eastern Nigeria (2, 3, 5, 6, 7).

The aetiology of PMS remains unknown but some factors attributed to nutrition such as amino acids, calcium, magnesium and vitamin B deficiencies; sex hormones as observed in the interplay of oestrogen and progesterone, also the excitatory and inhibitory roles of both serotonin and gamma-aminobutyric acid (GABA) are possible parameters for PMS development (4, 8). Other PMS factors include genetic makeup such as homology shared between mother and daughter as well as in twins; personality traits such as low self-esteem, introversion and extroversion; stress related to physical activity involving the stress hormone cortisol; use of oral contraceptive pills (OCPs); age; ethnicity and smoking (9, 10, 11, 12).

Manifestations of PMS include mood swing, irritability, depression, anger, anxiety, insomnia, breast tenderness, change in appetite, food craving, muscle pain, tinnitus, fatigue, oedema, bloating, social withdrawal, absenteeism from work/school, and poor work/academic performance (9, 13, 14, 15, 16). PMS management include administration of antidepressants like selective serotonin reabsorption inhibitors (SSRIs); others include non-steroidal anti-inflammatory drugs (NSAIDs) and OCPs, while non-drug management include intake of mineral supplements such as calcium and magnesium, intake of vitamins A, E and B_6_, exercising, yoga, and stress reduction programs (2, 4, 17). However, healthcare-seeking behaviour is poor as reports showed that many young adolescents and women suffering from dysmenorrhea either ignore the pain and suffer in silence or practice self-medication (18, 19, 20). This study aimed to elucidate the effect of mood swing, confounding factors, and healthcare-seeking behaviours of women during menstruation in an educational environment.

## Methods

**Selection of participants**: This descriptive cross-sectional study was conducted in an educational environment between February and June, 2018 in Delta State, Nigeria. Three hundred and twenty-eight women within reproductive ages of 16 and 35 years participated in the study. Participants' consents were sought; women with amenorrhea, recent debilitating disease, psychological problems, or using contraceptives were all excluded from the study.

**Sample size determination**: This was done according to Akoku and Minichil (2, 3) with little variation using the single population formula by adopting the estimated proportion of 69.2% mood swing prevalence from a previous study, using 95% Confidence Interval (α=0.05), standard normal distribution Z at 1.96, 5% margin error. Exactly 328 participants were considered for this study.

**Data collection**: A survey method was adopted; a validated and standardized questionnaire was administered to confidentially assess the effect of PMS, confounding factors and healthcare-seeking behaviour of women during menstruation. The questionnaire was grouped into four constructs containing seventy-nine questions. The first assessed demographic data such as age, marital status, type of family, religion, education and occupation; the second assessed reproductive health history such as endometriosis and other gynaecological challenges; third construct assessed mood swing via PMS, PMDD and menstrual cycle patterns, and the last assessed confounding factors and treatment history.

**Statistical analysis**: All data were analysed with SPSS 25.0 where descriptive method was adopted and results were expressed in percentages (%). The questionnaire's reliability testing was done using Cronbach's alpha and a value of 0.7 or higher was considered reliable. The overall frequencies were expressed as the mean frequencies in percentages (%).

**Ethical clearance**: The ethical clearance was obtained from the Research and Ethics Committee of the Faculty of Basic Medical Sciences (DELFBMS/0234).

## Results

The reliability test revealed Cronbach's alpha to be 0.863; the overall prevalence of PMS and PMDD were 67.4% (221) and 25.6% (84) respectively as shown in Table 1.

**Table 1:** The Prevalence of Premenstrual Symptoms and Premenstrual Dysphoric Disorders in Women (N=328)

	Mean Frequency n (%)						Total n (%)	
			
Overall Symptoms	Pscho-emotional		Physical		Behavioral			
	Yes	No	Yes	No	Yes	No	Yes	No
PMS	224 (68.3)	104 (31.7)	241(73.5)	87 (26.5)	197(60.1)	131(39.9)	221(67.4)	107(32.6)
PMDD	89 (27.1)	239 (72.9)	79 (24.1)	249(75.9)	85 (25.9)	243(74.1)	84 (25.6)	244(74.4)

PMS, Premenstrual Symptoms; PMDD, Premenstrual Dysphoric Disorder

**Figure 1:**
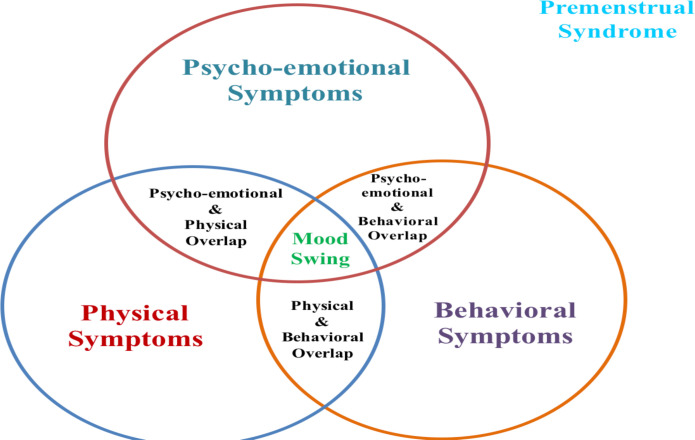
A Venn diagram showing mood swing as an overlapping symptom in PMS during menstruation in women.

Also, 22.9% (75) had a family history of bipolar disorder, while 30.2% (99) had previous diagnosis of bipolar disorder as shown in Table 2. Mood swing was discovered as a symptom overlapping psycho-emotional, physical, and behavioral symptoms (Figure.1), as these symptoms are relatively associated. Psycho-emotional symptoms include: anger, frustration, and irritability, 81.4% (267); feeling flat and depression, 73.2% (240), and easy distractions, 50.3% (165). Physical symptoms include: coldness, fatigue and/or paleness, 74.4% (244), food craving 76.5% (251); breast tenderness, abdominal and/or pelvic pain, 80.2% (263); fluid retention, abdominal bloating 70.7% (232). Other symptoms were dull, diffuse headache 75.9% (249), and premenstrual migraine or tension headache 66.5% (218). Furthermore, 71.0% (233) experienced diarrhea, constipation, and/or digestive changes while 72.9% (239) experienced worse PMS episodes during stress. Behavioral symptoms include: social withdrawal during the day and/or experience nocturnal social activities, 81.4% (267); absenteeism from work or school and/or poor work or academic performance, 73.2% (240); increased libido, 43.5% (143) and irrational behavior 42.4% (139).

**Table 2:** History and Previous Diagnosis of Bipolar Disorders (N=328)

History and Previous Diagnosis of Bipolar Disorder	Participants N(%)	Response N(%)

	Yes (%)	No (%)
Previous diagnosis of bipolar disorder	99 (30.2)	229 (69.8)
Family history of bipolar disorder	75 (22.9)	253 (77.1)

Results in Table 3 showed that gynaecological conditions ranged from endometriosis, 12.5% (41); uterine fibroid, 14.9% (49); ovarian cyst, 14.0% (46); pelvic adhesion, 12.8% (42) and polycystic ovarian syndrome, 14.0% (46).

**Table 3:** Gynecological Health Related Conditions in Study Subjects **(N=328)**

Gynecological Conditions	Likert Scale	Frequency (%)
Endometriosis	Yes	41 (12.5)
	No	187 (57.0)
	Not Sure	55 (16.8)
	Not Applicable	45 (13.7)

Uterine Fibroid	Yes	49 (14.9)
	No	179 (54.6)
	Not Sure	58 (17.7)
	Not Applicable	42 (12.8)

Ovarian Cyst	Yes	46 (14.0)
	No	183 (55.8)
	Not Sure	56 (17.1)
	Not Applicable	43 (13.1)

Pelvic Adhesion	Yes	42 (12.8)
	No	183 (55.8)
	Not Sure	59 (18.0)
	Not Applicable	44 (13.4)

Polycystic Ovarian Syndrome	Yes	46 (14.0)
	No	180 (54.9)
	Not Sure	57 (17.4)
	Not Applicable	45 (13.7)

The overall pain prevalence was 69.5% (228) experienced at different threshold (Table 4), where 22.6% (74) were regular, 29.6% (97) occasional, 17.4% (57) rare while 30.4% (100) never experienced pain. Subjects experienced downward, dragging sensation in the abdominal region, 78.0% (256); sharp pain accompanied with nausea and/or strong pelvic cramping, 75.9%; pain soothed by warmth and pressure, 72.3% (237); sharp, stabbing pain that only subsides when lying down, 66.2% (217); mild or moderate pain accompanied with diarrhea or lose stool, 50.3% (165); severe pain accompanied with fainting or vomiting, 61.3% (210); pain which subside when not under stress, 68.6% (225).

**Table 4:** Overall Premenstrual Pain (N=328)

Symptoms	Likert Scale	Frequency (%)	Total n (%)
Overall Premenstrual Pain Experience	Always	74 (22.6)	228 (69.5)
	Occasionally	97 (29.6)	
	Rarely	57 (17.4)	100 (30.4)
	Never	100 (30.4)	

As shown in Table 5, 60.1% (197) used medications for PMS; 14.3% (47) were regular users, 29.9% (98) occasional users, 15.9% (52) rarely use drugs while 39.9% (131) never used drugs. Among those that used medications, 17.7% (58) were always relieved, 23.5% (77) sometimes got relieved, 8.2% (27) rarely got relieved, while 10.7% (35) were never relieved of pain.

**Table 5:** The treatment behavior of women during PMS (N=328)

Treatment Behavior	Likert Scale	Frequency (%)
Used drugs during menstruation	Always	47 (14.3)
	Occasionally	98 (29.9)
	Rarely	52 (15.9)
	Never	131 (39.9)

Pain relieved after taking pain medication	Always	58 (17.7)
	Occasionally	77 (23.5)
	Rarely	27 (8.2)
	Never	35 (10.7)

Moreover, 26.5% (87) used Paracetamol; 14.3% (47) used Piroxicam; 4.3% (14) used Ibuprofen; 3.0 % (10) used Ladinax; 2.7% (9) used Aspirin while 9.1% (30) used other pain medications (Table 6). Reasons include headache, 13.7% (45); paleness, 22.3% (73); and pain, 24.1% (79).

**Table 6:** Choice of medication during PMS and reason for taking them (N=197)

	Name of pain reliever	Frequency (%)
Choice of pain medication	Paracetamol	87 (26.5)
	Aspirin	9 (2.7)
	Ibuprofen	14 (4.3)
	Ladinax	10 (3.0)
	Piroxicam	47 (14.3)
	Others	30 (9.1)

	**Symptom(s)**	**Frequency (%)**

Reason for using drug	Headache	45 (13.7)
	Paleness	73 (22.3)
	Pain	79 (24.1)

## Discussion

In the present study, the observed Cronbach's alpha (0.863) was greater than 0.7 standard statistical values, indicating that the instrument was valid and reliable. Also, Mood swing was discovered as a symptom overlapping with psycho-emotional, physical, and behavioural symptoms in women during menstruation, suggesting that these symptoms are relatively associated. The overall PMS prevalence was 67.4% which was slightly higher than but comparable to 67% reported from Ethiopia (7), lower than 77.8% and 78.6% reported from Cameroon and Eritrea respectively (3, 21), far below 89%, 91.5%, 92% and 99.5% prevalence reported somewhere in Nigeria, Jordan, Pakistan and Japan respectively (8, 11, 22, 23). This variation may be due to differences in the population samples, instruments used, PMS pattern, other factors like stress induced hypertension, and side effects associated with OCP usage; a potential association between high PMS prevalence and women using OCPs has been reported in one study (3).

The recorded array of prevalence of psycho-emotional symptoms physical and behavioural symptoms in the present study agrees with other reports from Saudi Arabia, Nigeria, and Japan respectively even though, they were categorized differently (1, 8, 12, 22).

The observed 25.6% PMDD prevalence was lesser, but comparable to 34.7% prevalence reported from Ethiopia, while it was far below 47.6% and 61% reported from Nigeria and Japan respectively (4, 11). This discrepancy might be attributed to underlying conditions such as psychiatric disorders, population studied, geographical distribution and methods used.

Moreover, 30.2% had previous diagnosis of bipolar disorder (BD) while 22.9% had a family history of bipolar disorder, suggesting a critical factor for mood swing, which may be aggravated by menstrual cycle. This finding agrees with a review among females with BD where 45% to 68% experienced premenstrual mood symptoms while women with PMDD have higher chances of bipolar disorder diagnosis (24). Another review showed that BD-PMDD comorbidity was reported in 70% cases, although PMDD can be observed in women without psychological disorders. The authors reported 10% prevalence of BD in women with PMDD which is seven-folds higher than those without PMDD, suggesting a strong link between BD and PMDD (25).

In this study, stress was observed to contribute to high prevalence as studying or working in academic/clinical environment comes with associated stress in Nigeria (9, 11, 22). A report on the magnitude of PMDD and its correlation with academic performance among medical students in Ethiopia corroborates that stress, severe menstrual pain and its negative impact on academic performance are associated with PMDD; our study and other reports from Japan and Nigeria respectively agree with this discovery (2, 4, 11, 19). However, interference of PMS or PMDD with academic or work performance was not reported in some studies (1, 11).

The findings that 12.5% participants had endometriosis, 14.9% had uterine fibroid, 12.8% had pelvic adhesion, while 14.0% had ovarian cyst and polycystic ovarian syndrome respectively, suggests that there may be an association between these gynaecological conditions and premenstrual symptoms like anxiety, dysmenorrhea, menorrhagia, abnormal vaginal bleeding and pain threshold which may trigger the healthcare-seeking behaviour in women. Endometriosis usually presents symptoms such as menstrual bleeding (menorrhagia), painful periods (dysmenorrhea), painful intercourse (dyspareunia), painful defecation (dyschezia), painful urination (dysuria) as well as pelvic pain, all of which vary in women, and largely agrees with this study (26). Furthermore, The American College of Obstetricians and Gynaecologists identified endometriosis, ovarian cysts, pelvic inflammatory disease, and pelvic adhesions as culprits in dysmenorrhea which corroborates the findings in this study (27).

Severe pain and its interference with academic/work performance was observed in to be the major reason for seeking healthcare. The overall pain prevalence was 69.5%, this agrees with 68.7%, 66.9%, and 73.6% previously reported from China, India and Ethiopia respectively, another study reported that nearly two-thirds with chronic acyclic pain undergoing laparoscopy had endometriosis (2, 27, 28, 29). Moreso, while 60.1% opted for medications, 39.9% were relieved of pain when not under stress, so they did not seek medical treatment. The 60.1% and 39.9% obtained respectively were higher, but comparable to 56% and 24% reported from India respectively. This does not concur with the report from Ogun State, Nigeria where 46.3% endured dysmenorrhea while 29.3% used over the counter drugs, and another from China where 62.5% girls with dysmenorrhea did not take medications and 31.6% took medication (19, 28, 30).

Reasons for taking drugs include headache (13.7%), paleness (22.3%) and other types of pain (24.1%). Paleness, as observed in this study suggests that some women may have heavy menstruation resulting into severe anaemia. Gynaecological conditions as well as genotype of the patient may account for increased predisposition of women to severe anaemia during menstruation.

The frequency of most commonly used pain medications [Paracetamol (26.5%) and Piroxicam (14.3%) were frequently used, followed by Ibuprofen (4.3%)] concur with previous studies (19. 28. 30). However, not all subjects who used pain medications got relieved and this may be due to the choice of drug, underlying health conditions such as paleness associated with anaemia, financial ability, the educational and/or professional background of the patient.

In conclusion, PMS and PMDD prevalence among young girls and women is relatively high. Mood swing was observed as a symptom overlapping with psycho-emotional, physical and behavioural symptoms. Underlying diseases such as bipolar disorders, previous history of bipolar disorder and gynaecological diseases like endometriosis, uterine fibroid, ovarian cysts and post cystic ovarian syndrome are the major culprits for mood swing thereby causing the exacerbation of PMS resulting into PMDD. Additionally, stress is another confounding factor aggravating PMS in both women with or without underlying conditions, resulting in PMDD. Consequently, PMDD can trigger mood swing and can badly affect academic/work activities when accompanied with severe pain. Victims either ignorantly endure the pain due to socio-cultural and financial factors or take unsuitable medications and drug abuse is inevitable. Hence, there is need for the government and relevant institutions to educate young girls and women about menstruation, how to manage some of the symptoms and when to consult medical personnel.
